# Relapsed Refractory Hodgkin Lymphoma and Brentuximab Vedotin-Bendamustine Combination Therapy as a Bridge to Transplantation: Real-World Evidence From a Middle-Income Setting and Literature Review

**DOI:** 10.3389/fonc.2021.796270

**Published:** 2022-01-21

**Authors:** Vivek S. Radhakrishnan, Rajat Bajaj, Vasundhara Raina, Jeevan Kumar, Saurabh J. Bhave, Reghu K. Sukumaran Nair, Arijit Nag, Indu Arun, Lateef Zameer, Debdeep Dey, Neeraj Arora, Mayur Parihar, Jayanta Das, Rimpa B. Achari, Deepak K. Mishra, Mammen Chandy, Reena Nair

**Affiliations:** ^1^ Clinical Haematology Oncology and Haematopoietic Cell Transplantation (HCT), Tata Medical Center, Kolkata, India; ^2^ Paediatric Haematology Oncology, Tata Medical Center, Kolkata, India; ^3^ Histopathology, Tata Medical Center, Kolkata, India; ^4^ Laboratory Haematology Cytogenetics and Molecular Pathology, Tata Medical Center, Kolkata, India; ^5^ Nuclear Medicine, Tata Medical Center, Kolkata, India; ^6^ Radiation Oncology, Tata Medical Center, Kolkata, India

**Keywords:** Hodgkin lymphoma, relapsed/refractory, Brentuximab vedotin (Adcetris) ^®^, bendamustine, transplantation, real world data (RWD)

## Abstract

**Introduction:**

Despite high cure rates with standard treatment, 30% patients with Hodgkin lymphoma develop relapsed or refractory (R/R) disease. Salvage therapy followed by autologous hematopoietic cell transplantation (HCT) is considered standard of care. Brentuximab Vedotin (Bv) in combination with Bendamustine (B) has been tested in the salvage setting with promising results.

**Materials and Methodology:**

We conducted a single centre retrospective chart review of patients who received BBv salvage therapy to determine its activity and safety in patients with R/R classical Hodgkin lymphoma (HL). Between May 2011- December 2019, 179 patients were diagnosed with R/R HL.

**Results:**

Thirty patients received BBv [median age: 30 (15-59) years, females (n=15)]. Primary refractory disease in 19 patients (63%), and 26 patients (87%) had advanced stage at treatment. Most patients received BBv after 2 prior lines of therapy [n=16 (53%)]. The median number of cycles of BBv were 3 (1-6). The number of BBv cycles delivered as outpatient was 63%. The most common Grade III/IV hematological adverse event was neutropenia [n=21, (70%)], while grade III/IV non-hematological toxicities included infections in 4 (13%), neuropathy in 4(13%), skin rash in 2 (7%), GI toxicities in 3 (10%) and liver dysfunction in 2 (7%) patients. The ORR and CR rates were 79% and 62%, respectively. Seventeen patients (57%) underwent an autologous HCT and 8 (26%) underwent an Allogeneic HCT (all haploidentical). The median follow up time from BBv administration was 12 months. Six patients died: 2 = disease progression, and 4 = non-relapse causes (Infection and sepsis = 2, GVHD=2). In addition to this, one patient progressed soon after HCT and another patient relapsed 22 months post HCT. Three year Overall survival (OS) and Event free survival (EFS) probability post-BBv treatment was 75% and 58%, respectively. OS and EFS analysis based on response (viz., CMR) to BBv demonstrated that patients in CMR had better survival probability [93% (p=0.0022) 3yr-OS and 72% (p=0.038) 3yr-EFS probability].

**Conclusions:**

BBv is an active and well-tolerated salvage treatment for patients with R/R HL, even in refractory and advanced settings. In middle-income settings, cost constraints and access determine patient uptake of this regimen.

## Introduction

Hodgkin lymphoma (HL) is a lymphoproliferative disorder with a crude incidence of 2.3 cases/100,000 individuals, and a bimodal age distribution affecting young adults (aged 15–30 years) and a second peak occurring in individuals aged > 55 years (1, 2). The hallmark of HL is the presence of Reed-Sternberg cells, and these cells consistently express the cell membrane receptors CD30 and CD15, demonstrated by immunohistochemistry. HL has a very good prognosis, with more than 75% of patients experiencing a clinical cure after front-line therapy (2). However, in early and advanced stage disease, 5% and 30–40% of patients, respectively, relapse after front-line therapy (3). The standard of care for patients with relapsed or refractory disease is salvage chemotherapy followed by high-dose chemotherapy (HDCT) and autologous haematopoietic cell transplant (AHCT) in the transplant-eligible. Only 50% of patients are cured with standard salvage therapies (1–4). Achievement of a complete metabolic response (CMR) prior to an AHCT is associated with significantly better outcomes (5). Patients who relapse after salvage HDCT/AHCT generally have a poor overall outcome and dismal long-term prognosis (6).

Brentuximab Vedotin (Bv), is a targeted monoclonal antibody-drug conjugate (ADC) active against CD30-positive cancer cells, such as those associated with classical Hodgkin lymphoma or Anaplastic large cell lymphomas (ALCL) (7, 8). CD30 is an ideal target for ADC based therapy because it has high levels of expression on tumor cells in HL and demonstrates limited expression on normal B or T cells (8). Bv is a chimeric CD30-specific immunoglobulin (Ig) G1 antibody conjugated to the microtubule disrupting agent, monomethyl auristatin E (MMAE), by an enzyme-cleavable linker. MMAE binds to tubulin, resulting in cell cycle arrest and subsequent apoptosis of CD30-expressing cells (9, 10). Bv has been approved for use in relapsed (in 2011) and newly diagnosed (in 2018) Hodgkin Lymphoma recently (11). In 2008, mouse models showed that BV could be combined with chemotherapy with augmented anti-tumor efficacy (12). Bendamustine, a dual functional alkylating chemotherapeutic agent with a benzimidazole ring (purine analog), has limited cross-resistance with other alkylators. Bendamustine has been shown to be effective in patients with relapsed HL including those post-Bv therapy, possibly indicating its role in overcoming Bv resistance (13, 14). An *in-vitro* study has shown that bendamustine increased CD30 expression on tumor cells, thereby bringing forth a “proof-of-concept” of their synergism in relapsed or refractory (R/R) HL (15). A trial was initiated in 2012 exploring the combination of Bendamustine and Brentuximab vedotin (BBv) on the premise of prior phase 2 monotherapy studies, and early results were reported in 2014 which brought into limelight a new salvage regimen for the R/R HL cohort (16). The final results of this study and two other prospective phase 1/2 trials using BBv in the salvage setting have demonstrated encouraging response rates, a manageable toxicity profile and successful stem cell mobilization (17–19).

There is limited real-world evidence in terms of activity and tolerability of Bv or Bv based combination therapies in the relapsed or refractory (R/R) HL setting (20, 21). Our centre began using this combination from 2012 in refractory and relapsed Hodgkin lymphomas, based on the evidence available, expert opinion, institutional consensus, and named-patient regulatory permissions in the setting of patients with R/R HL disease, who had no access to clinical trials or alternative therapies (12–15). This retrospective study is a single center audit of the real-world efficacy and tolerability of the combination Brentuximab vedotin and Bendamustine (BBv) regimen in R/R Hodgkin lymphoma patients, either as first salvage or in subsequent lines of therapy, from a middle-income country setting.

## Materials and Methods

### Study Design

This study is a single centre, retrospective observational study, of R/R HL patients treated with BBv in a tertiary care cancer center in India. The hospital electronic medical records (EMR) were used to identify all patients, 15 years or older treated with BBv for R/R HL from 2011, as part of their salvage chemotherapy. The diagnosis of Hodgkin lymphoma was based on biopsy performed at diagnosis and/or at relapse. Refractory disease was defined as Deauville score > 3, after the completion of first line multi-agent chemotherapy. Routine staging procedures at time of diagnosis and at relapse or progression included a full history and physical examination, routine laboratory investigations, computed tomography scanning of the neck, thorax, abdomen, and pelvis with bone marrow biopsy or Fluorodeoxyglucose–positron emission tomography Computerized axial tomography (FDG-PET-CT) scan. Bone marrow examination was not done for patients, in whom FDG-PET-CT scan was done. In patients who had clinical progression, no additional imaging was performed. All the R/R patients were offered BBv, as first line salvage therapy. However, in view of financial and logistical constraints, not all of them received BBv as their first salvage treatment. These patients received other approved salvage chemotherapy protocols like Cisplatin, Dexamethasone and Cytosine arabinoside (DHAP), Gemcitabine, cisplatin and dexamethasone (GDP) or Ifosfamide, Carboplatin and etoposide (ICE).

Combination chemotherapy included Bv at a dose of 1.8 mg/kg, as intravenous infusion on day 1 along with Bendamustine at a dose of 90 mg/sq.m on days 1 and 2 of a 21-day cycle. Patients received standard corticosteroid (dexamethasone 4-8mg), antihistaminic (diphenhydramine or chlorpheniramine) and antiemetic (ondansetron) premedication. The first infusions were administered inpatient while the rest were delivered in the out-patient daycare service for all patients, except the first patient. Response evaluation with FDG-PET-CT scan was done at the end of 2-3 cycles, based on the international working group (IWG) revised response criteria for malignant lymphoma, and from 2015 the Lugano classification (22, 23). The patients who were ≥ partial response (PR), and willing for transplant were eligible for consolidation with AHCT or Allogeneic Hematopoietic cell transplantation (alloHCT). Transplant-ineligible patients received up to 6 cycles with an intent to achieve a durable remission. Growth factors (e.g., filgrastim) were used routinely as primary prophylaxis in most, to maintain planned dosing schedules. The patients who were < PR, after 3 cycles of BBv, were given additional or alternative chemoimmunotherapy, including check point inhibitors. Treatment toxicity and treatment related mortality were audited. We also reviewed literature from indexed peer-reviewed publications citing Bv based regimens as well as Indian data, in R/R HL. This study received a waiver from the Institutional review board for anonymized data analysis.

### Response Assessment and Study Endpoints

The patients who received at least 2 cycles of BBv were considered evaluable for response, toxicity, and efficacy. The primary endpoint of the present study was the overall response rate (ORR), which included complete metabolic response (CMR) rate and partial response (PR) rate, to the combination regimen. The data cut-off date was April 30, 2021. Secondary endpoints were determined by time to event analysis as overall survival (OS) and event free survival (EFS). EFS was defined as the time from initiation of BBv treatment to the first episode of relapse, progression, or death from any cause. OS was defined as the time from initiation of BBv treatment to the event of death from any cause. Safety assessment was performed in all patients receiving at least one dose of the combination regimen. The severity of adverse events (AEs) was graded according to NCI CTCAE (National Cancer Institute Common Terminology Criteria for Adverse Events), version 4.03 (24).

### Statistical Analysis

Patient demographics and other clinical diagnosis features were summarized by descriptive statistics. Median and range was computed for continuous variables, and categorical variables were reported as absolute figures and percentage frequencies. The Kaplan–Meier method was used to estimate the survival function with 95% CI using R version 4.0.2 (25). Survival curves were compared by p-value obtained from log-rank test. The threshold for statistical significance was considered as p ≤ 0.05.

## Results

### Patient Clinical Profile, Allocation to BBv Treatment and Toxicity Profile

Between 2011-2019, 833 patients were registered with a diagnosis of Hodgkin Lymphoma in our department. Among the 179 patients with R/R HL, a total of 30 patients with R/R Hodgkin lymphoma received BBv and were considered eligible for this retrospective study. The demographic and baseline clinical features of the patients at diagnosis are summarized in **Table 1**. The median age of the patients was 30 years (15-59 years) with 15 (50%) males. Only 4 patients had significant medical comorbidities, diabetes mellitus in 4 and hypertension in 1. ABVD was the most frequent (90%) primary (or first-line) regimen used in this group. The ORR to the respective first line therapies in this cohort was 50% (n=15), while 12 (40%) patients showed disease progression (PD) and 3 (10%) developed stable disease (SD). The patients who were in complete response (CR) relapsed early (<1 year), while the rest had PD. Nineteen patients received other second line salvage chemotherapy prior to BBv, and GDP was the most frequent regimen (n=14, 47%). Prior to BBv, 5 patients had undergone HCT (4 AHCT and 1 alloHCT) but relapsed early (<1yr). Most of the patients receiving BBv salvage (n=26, 87%) had advanced stage III-IV disease at the time of treatment and many had refractory disease (n=19, 63%). None of these patients had received Bendamustine or Brentuximab vedotin alone or in other combinations, as a treatment line prior to BBv.

**Table 1:** Patient demographic and clinical profile.

Features		Median no. of cycles (range)
**Median Age (range)**	30 (15–59)	
**Gender (%)**		
Male	15 (50)	
Female	15 (50)	
**Subtype (%)**		
Mixed cellularity	6 (20)	
Nodular sclerosis	6 (20)	
lymphocyte depleted	1 (3)	
Not otherwise specified/Unassigned	17 (57)	
**First Line Treatment, N=30 (%)**		
ABVD	27 (90)	6 (2-6)
ABVD/BEACOPP	1 (3.3)	
CHOEP	1 (3.3)	
OEPA	1 (3.3)	
**Second Line Treatment, N=22 (%)**		
GDP	14 (47)	3 (1-4)
DHAP	4 (13)	
ICE	3 (10)	
other	1 (3)	
**HCT pre BBv (N=5)**		
AlloHCT	1 (20)	
AHCT	4 (80)	
**Treatment lines prior to BBv, N=30 (%)**		
1	8 (27)	
2	16 (53)	
3	4 (13)	
4	2 (7)	
**Disease stage prior to BBv, N=30 (%)**		
II	4 (13)	
III	9 (30)	
IV	17 (57)	
**Bulky disease prior to BBv (%)**	4 (13)	
**Extra nodal disease prior to BBV (%)**	6 (20)	
**B symptoms prior to BBV (%)**	5 (17)	
**Median no. of cycles of BBv (range)**	3 (1-6)	
**HCT as consolidation, N=25 (%)**		
AHCT	17 (68)	
AlloHCT (Haploidentical donor)	8 (32)	
**Graft Source (%)**		
PB	25 (100)	
**Conditioning (%) N=25**		
BEAM	15 (60)	
Gem-Bu-Mel	2 (8)	
FT10	5 (20)	
FLU-Mel	3 (12)	
**Stem cell mobilisation (%)**		
GCSF alone	10 (40)	
GCSF + plerixafor	15 (60)	
**Median CD34+ cell dose x10^6^/kg (range)**	4.9 (2.3-9.3)	
**Median MNC x10^6^/kg**	9.7 (2.6-27.3)	
**Median day of engraftment (range)**		
Neutrophil (AHCT)	9 (7-11)	
Neutrophil (AlloHCT)	16 (15-21)	
Platelet (AHCT)	11 (7-15)	
Platelet (AlloHCT)	22 (16-31)	
**Outcomes (%)**		
Relapse		2 (6.6)
Death		6 (20)
**Regimen related toxicity, N=12 (%)**		
Grade 1		4 (33)
Grade 2		8 (66)

As elaborated in **Table 1**, BBv regimen was administered as second line treatment to 8 patients, third line to 16 patients and as fourth or subsequent lines in 6 patients. Median number of BBv cycles received was 3 (range 1-6). Only one patient received 6 cycles, without HCT. Grade I/II infusion reactions were noted in 12 patients (40%), however no grade III/IV infusion reactions or anaphylaxis were reported. Most infusions (63%) were delivered in out-patient service. The major Grade III/IV adverse events observed in the regimen included the following: neutropenia in 21 patients (70%) [28 of 92 cycles of BBv administered (30.4%)], mucositis in 3 patients (10%), peripheral neuropathy in 4 patients (13%), skin rash in 2 patients (6.5%), and clinically detected infection in 4 patients (13%). One patient developed febrile neutropenia and died due to septic shock after cycle 1 of BBv [and is excluded from response assessment and response-stratified survival analyses]. **Table 2** elaborates the toxicity profile. There were no therapy discontinuations or treatment delays due to BBv toxicity. Overall, it was a well-tolerated regimen.

**Table 2:** Treatment related adverse events.

Non-hematological toxicity (Grades 3 and 4)	N (%)
Infection	4 (27)
Rash	2 (13)
Neuropathy	4 (27)
GI	3 (20)
Liver/Hepatitis	2 (13)
**Haematological toxicity**	**N (%)**
III	21 (95)
IV	1 (5)

### BBv Treatment Response and Events

The response profile of patients (n=30) across different treatment lines [prior to BBv, post-BBv and post consolidation (HCT)] are described in **Table 3**. Post-BBv, an ORR of 79% (n=23) was observed, with a complete metabolic response (CMR) in 18 (62%) and PR in 5 (17%) patients. These patients were transplant eligible. One patient had SD and 5 patients (17%) had PD. Of the 5 patients who had PD: one patient died due to disease progression, while 2 received Nivolumab + Brentuximab vedotin + Lenalidomide, 1 received Nivolumab alone, 1 underwent radiation therapy (RT, 30.6 Gy) and achieved CMR. The lone patient with stable disease received Nivolumab + vinorelbine and achieved CMR. Five more patients thus became transplant eligible.

**Table 3:** Response assessment based on different treatment lines before BBv, post-BBv and consolidation.

Time points	Clinical Response to the Treatment				
	CMR	PR	SD	PD	ORR
Post 1^st^ line therapy (n=30)	11 (37%)	4 (13%)	3 (10%)	12 (40%)	15 (50%)
Post 2^nd^ line therapy (n=22)	4 (18%)	10 (46%)	2 (9%)	6 (27%)	14 (64%)
Post BBv (n=29)	18 (62%)	5 (17%)	1 (3%)	5 (17%)	23 (79%)
Response prior to HCT consolidation (n=25)	20 (80%)	5 (20%)	0	0	25 (100%)
Post HCT consolidation (n=23)	21 (91%)	0	0	2(9%)	21 (91%)

CMR, complete molecular response; PR, partial response; SD, stable disease; PD, progressive disease; ORR, overall response rate (CMR + PR).

### Stem Cell Mobilization and HCT

All patients with a response (CMR or PR) were counselled to undergo a consolidation HCT. Twenty-eight patients were transplant eligible. Two patients dropped out due to financial constraints and they remain in CMR at data-cutoff, over a follow-up period of 44.6 and 9.9 months each. 1 patient (who received RT to achieve CMR) failed autologous stem cell mobilization and remains in CMR. Mobilization regimen for all AHCT patients involved the use of both Granulocyte-colony stimulating factor (G-CSF) and plerixafor. Allogeneic donors underwent G-CSF mobilized peripheral blood stem cell apheresis.

Twenty-five patients underwent a HCT [20 post BBv, 5 after subsequent regimens]. Response status prior to HCT included 20 patients in CMR and 5 in PR. Patients undergoing AHCT and alloHCT were 17 and 8 (all Haploidentical donor), respectively. The patients who underwent alloHCT were essentially those who were heavily pre-treated or had chemo-refractory disease, pre-BBv [Relapsed-refractory (n=6) and primary refractory (n=2)]. Three of these patients were post AHCT (autologous HCT). Conditioning regimen used in AHCT was BEAM [BCNU (carmustine), Etoposide, Arabinoside cytosine, and Melphalan] (n=15) and Gem-Bu-Mel (Gemcitabine-Busulfan-Melphalan) (n=2). The median CD-34 cell dose in the AHCT group was 5.32 x 10^6^ (2.7-9.6) cells/kg. In the haploidentical alloHCT group, the conditioning regimens used were Fludarabine-Treosulfan (n=5), and Fludarabine-melphalan [n=3]. The median CD-34 cell dose in this group was 4.9 x 10^6^ (4.1-5.9) cells/kg. Graft-versus-Host-Disease (GVHD) prophylaxis used in alloHCT was post-transplant Cyclophosphamide (PTCy), and Tacrolimus-Mycophenolate mofetil (Tac-MMF) regimen in the Haploidentical alloHCT patients. Graft source in all patients was peripheral stem cell apheresis product. All AHCT patients received cryopreserved stem cell products, while haploidentical alloHCT patients after COVID-19 outbreak (March 2020) also received cryopreserved products, based on institutional practice. The median days to neutrophil and platelet engraftment were 9 and 11 days, and 16 and 22 days, in the AHCT and alloHCT patients, respectively.

### Post-HCT Events and Response

**Figure 1:**
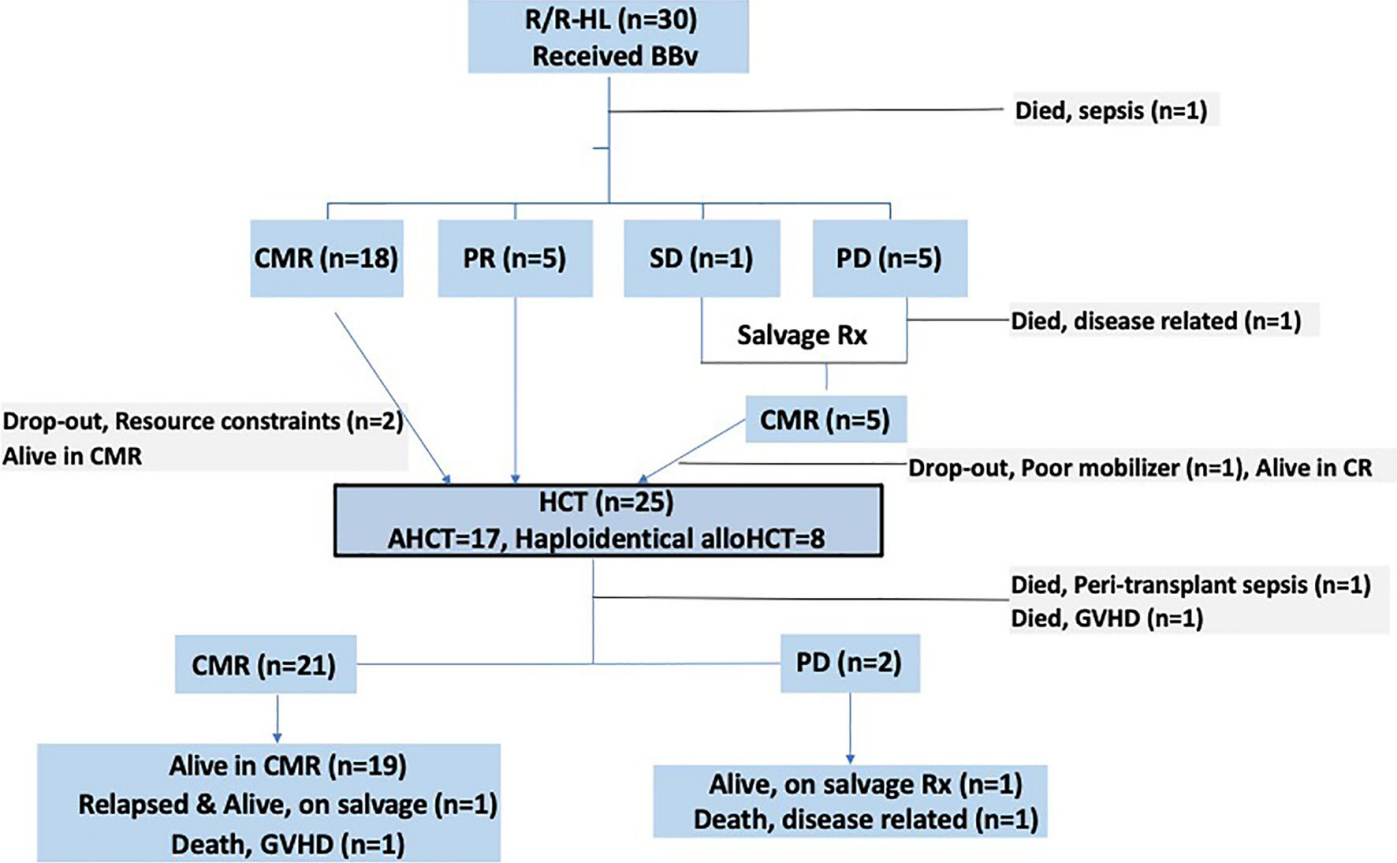
Patient flow diagram of the study.

Two haploidentical alloHCT patients died in the peri-transplant period, 1 each of sepsis and acute severe steroid refractory GVHD. Post-HCT response assessment was thus possible in 23 patients: 21 were in CMR while 2 developed PD. To elaborate, of the 5 patients who had a partial response prior to HCT, 3 achieved CMR post-HCT, 2 patients developed progressive disease. Of the remaining 18 patients (who had CMR pre-HCT), one haploidentical alloHCT patient died due to steroid refractory severe GVHD later and one patient developed a late relapse (22 months, is alive and in remission after sequential salvage therapies elsewhere). Of the two patients who had progression, 1 died and the other is alive (with disease) on salvage therapy. None of the surviving haploidentical alloHCT patients (n=5/8, 62.5%) have developed a relapse, at a median follow-up of 14.4 months (CI 95%: 6-23). Patient flow diagram in **Figure 1** provides a snapshot of this study.

### Survival Analysis

The median follow-up time from the date of initiation of BBv regimen was 12 months (95% CI: 5.2-18.8). Of the 30 patients, 6 died: 2 due to disease progression, and 4 due to non-relapse mortality (Infection and sepsis = 2, GVHD=2). Death due to disease progression/relapse occurred at 3.8 months and 10.8 months from BBv. Non-relapse deaths occurred at 3 weeks (sepsis post cycle 1 BBv), 8.7 months (acute severe steroid refractory GVHD), 10.5 months (sepsis), and 11.3 months (steroid refractory severe GVHD and sepsis) from BBv. Post-BBv treatment: 5 patients had progressive disease, one patient had persistent/stable disease, and 2 patients relapsed post-HCT.

**Figure 2:**
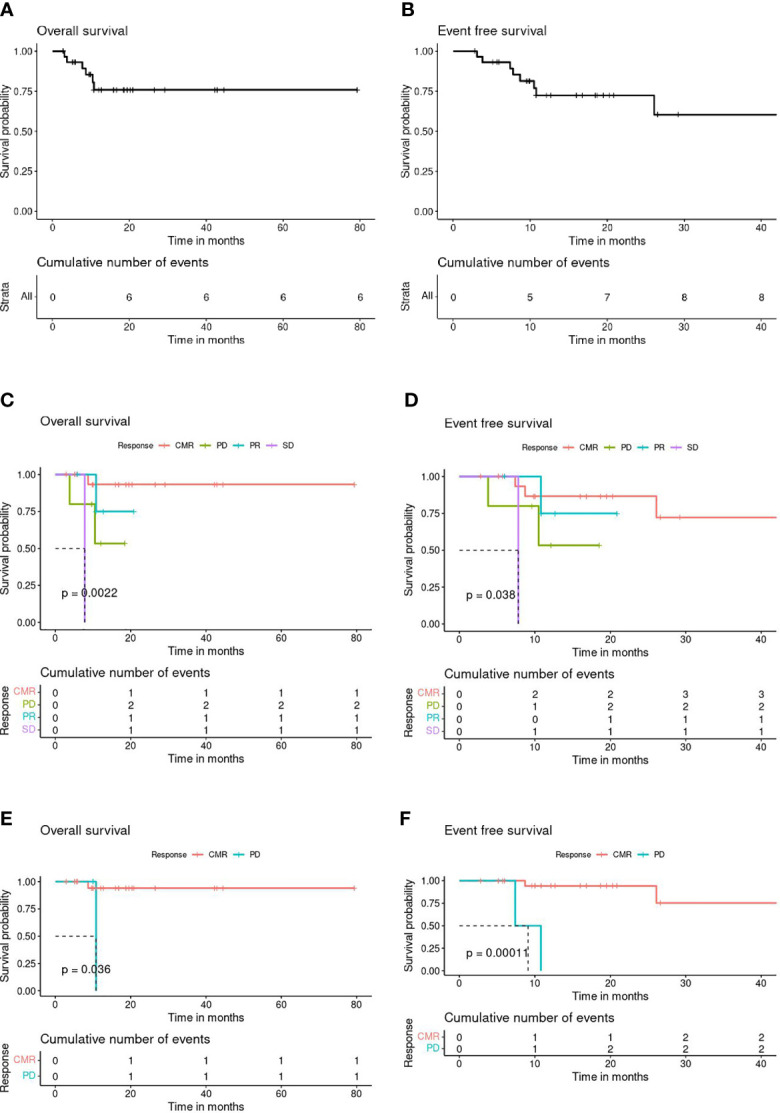
**(A)** Overall Survival (OS) all patients (n=30). **(B)** Event-Free Survival (EFS) all patients (n=30). **(C)** Post-BBv Response stratified OS (n=29). **(D)** Post-BBv Response stratified EFS (n=29). **(E)** Post-HCT Response stratified OS (n=23). **(F)** Post-HCT Response stratified EFS (n=23).

Overall survival (OS) and event-free survival (EFS) probability at 3 years post-BBv were 75% (CI: 59%-95%) and 58% (CI: 37%-90%), respectively (**Figures 2A, B**). Survival curves based on the clinical response to the BBv treatment demonstrated that the patient group achieving CMR post-BBv had better survival probability, OS - 93% [CI: 81.5%-100%] and EFS - 72% [CI: 48%-100%] at 3 years (**Figures 2C, D**). The median survival probability for patients with PD status was 10.5 months and 8.2 months for OS and EFS, respectively.

**Figures 2E, F** demonstrate the survival curves based on response assessed post HCT as consolidation, the CMR patient group showed significantly better survival probability at 3 years than patients with progressive disease after consolidation transplant: OS - 94% (CI: 84%-100%, p=0.033) and EFS - 72% (CI: 45%-100%, p=0.0018).

We independently evaluated a cohort of patients with R/R HL who did not receive BBv based salvage regimen prior to undergoing AHCT, in CR/PR, during the same period. Eighteen patients underwent AHCT (details in **Table S1**). The median age for this group was 25 years (16-50 years), with 11 (65%) males and 7 (35%) females. Salvage regimen GDP ± R was received by 9 (50%) patients, DHAP ± R by 7 (39%), GCD by 1 (5.5%), and IGEV by 1 (5.5%). Nine (50%) patients received these salvage therapies as 2nd line and the remaining as 3rd line. CMR was noted in 15(83%) patients while 3 (17%) attained PR. Post-AHCT, response information was available for 16 patients: 12 (74%) were in CMR while 4 (25%) progressed. In total we observed 7 events (38.5%) post AHCT (refer to outcomes in **Table S1**). At a median follow up of 20 months (CI 95%: 12-28), the OS and EFS estimate at 3 years were 76% and 51%, respectively (**Figures S1A, B**) as compared to the OS (80%) and EFS (60%) for the BBv cohort that underwent any HCT (Allogenic or AHCT) (n=25, **Figures S2A, B**), and OS (92%) and EFS (56%) for the BBv cohort that underwent only an AHCT (n=17, **Figures S2C, D**).

## Discussion

Patients with relapsed or refractory Hodgkin lymphoma are a difficult to treat subgroup and represent a felt need for improvisation of therapeutic approaches. In India, recent consensus recommendations advise the use of Bv or Bv based regimens in R/R HL, in second or subsequent lines of therapy (26). Brentuximab vedotin is not yet approved for regular prescription use and sale in India at the time of manuscript preparation. After its initial FDA approval in 2011, our group used Bv on a ‘named-patient licensed drug access’ program by importing the medicine for needy patients who could afford the expenses. From the same year, the combination of BBv was used by our group in the R/R HL patients. The per-capita GDP of India (2019 est.) is $6,700 (27). The current cost of one vial of Bv (50mg) is around $3429-3909 (28, 29) and does not include the markup (by the importing agencies) for logistics and handling costs. Currently, there are no comprehensive universal health insurance schemes covering complete cancer care in India and most treatment costs are borne out-of-pocket by patients or their caregivers. Even in developed economies with universal health coverage, the cost-effectiveness of Bv is a subject of active debate (30). In this background, it is not surprising that the uptake of a Bv based regimen in our institution was only 15-20% among eligible patients and reflects the constraints of practice in a LMIC (low- and middle-income country) setting.

There is a paucity of data from India on the management of patients with R/R HL. A perusal of peer-reviewed literature yielded only 3 significant studies (31–33) and this is elaborated in **Table 4**. These 3 recent retrospective studies provide an insight into the use of conventional chemotherapies in different, standard as well as novel, combinations in the in-patient setting. Between 24-74 patients were treated in each of these series, most with prior exposure to at least 1-2 salvage therapies, and achieved ORRs in the range of 63-79% [CR: 24-42%]. Nearly 45-92% patients underwent HCT, all AHCT. The OS and EFS rates varied between studies, with the best 3-year outcomes being 79% and 46%, respectively. As expected, a significant number of patients experienced grade-III/IV febrile neutropenia and chemotherapy induced nausea-vomiting in the salvage regimen. These studies also confirm that novel therapies have not yet made any significant entry in the treatment armamentarium of R/R HL in India and reflect a fact on the patterns of care in most middle-income countries.

**Table 4:** R/R HL-Chemotherapy salvage and AHCT, Indian studies.

Author	Year	Type of study	No. of pts	Median prior lines of treatment	Regimen	Prior AHCT	Median cycles	Response	Transplant (%)	Grade III-IV Tox (>10% patients)	EFS, %	OS, %
Prakash et al. [SC] (31),	2021	Retrospective	24	2	Vinorelbine Ifosfamide Bendamustine Etoposide (VIBE), q21d	no	3	ORR=79%, CR=42%	92%	Febrile Neutropenia Nausea Vomiting	46% at 3yrs	79% at 3years
Ganesan et al. [SC] (32),	2019	Retrospective	48	1	Gemcitabine, Vinorelbine, Dexamethasone (GVDex), q21d	no	3	ORR=63%	45%	Febrile Neutropenia	49% at 2yrs	60% at 2years
Raut et al. [SC] (33),	2016	Retrospective	74	3	Multiple (DHAP, ICE, etc.)	no	2-5	ORR=78%, CR=24%	51%	Transplant outcomes Transplant related mortality=10%	Transplant outcomes, DFS=65% at 5yrs	Transplant outcomes, OS=70% at 5yrs

SC, Single centre; ICE, Ifos + Cisplatin + Etoposide; DHAP, Dexamethasone, high dose ara-C, cisplatin; NR, Not reported/Not available; ORR, Overall response rate; CR, Complete response; PR, Partial response; EFS, Event free survival; OS, Overall survival.

The introduction of Bv and the inroads it has made in the treatment of HL is testimony to the rapidly evolving role of targeted immunotherapies of cancer. The use of Bv monotherapy and Bv based regimens in R/R HL has been growing in the last one decade. **Table 5** describes some of the significant studies in this setting (17–21, 34–41). The combination of BBv has been tested in the developed world in at least 3 prospective trials and more retrospective real-world studies (17–20, 39, 41). O’Connor et al. prospectively evaluated BBv in a phase 1/2 study in a heavily pre-treated subgroup of R/R HL patients and achieved an ORR of 78% [CR: 66%] and impressive survival outcomes (19). Subsequently, two groups tested the combination prospectively in the first-line salvage setting and achieved ORR in the range of 84.2-92.5% [CR: 78.9-73.6] (17, 18). Nearly 78-92% of these patients, in both studies, underwent AHCT. The EFS and OS at 3 years were in the range of 67.3% and 88.1%, respectively (18). Peripheral neuropathy and neutropenia were the commonly reported grade III/IV toxicities with an incidence of 10% or more in all the studies. The retrospective studies also used BBv in earlier lines of salvage therapy (first-line or second line salvage) and achieved ORR in the range of 79-92.6% [CR: 49-70.7%] (20, 41). Between 67-90% patients in both studies underwent AHCT. Lannitto et al. reported a median EFS of 18 months and 2-year OS of 72%, while Pinczés et al. reported 2-year EFS and OS outcomes of 62% and 93% respectively. The most common grade-III/IV toxicities reported in both the studies were neutropenia, gastrointestinal toxicity and neuropathy. Treatment discontinuation was infrequent. In most of these studies, achievement of a negative PET-CT scan following salvage therapy and proceeding to an AHCT significantly contributed to an improved EFS. The role and effect of post-AHCT maintenance Brentuximab (42), though recommended by different guidelines, could not be assessed in these studies due to the small sample size and the confounding effect of its pre-AHCT usage.

**Table 5:** Selected studies with Brentuximab based treatment in R/R HL.

Author	Year	Type of study	No. of pts, median age (yrs)	Median prior lines of treatment	Regimen	Prior AHCT	Median cycles	Response			Transplant (%)	Grade III-IV Tox (>10% patients)	EFS, %/median (months,m)	OS, %/median (months,m)
								ORR (%)	CR (%)	PR (%)				
Younes et al. [MN] (34),	2012	Prospective phase II	102, 31	3.5	Bv	Yes	16	75	34	41	NR	Neutropenia	5.6m	22.4m
Chen et al. [MC] (35),	2015	Prospective Phase II	37, 34	1	Bv	No	4	68	35	33	89	None	NR	NR
Cassaday et al. [SC] (36)	2016	Prospective	16, 32	1	Bv + ICE	No	3	94	88	6	75	Neutropenia	NR	NR
O’Connor et al. [MC] (19),	2018	Prospective Phase I/II	65, 34	Phase-1: 5 Phase-2: 3	Bv + Be	No	3	78	66	12	NR	Neutropenia	Phase-2: not reached	Phase-2: not reached
LaCasce et al. [MC] (17),	2018	Prospective phase I/II	49, 36	1	Bv + Be	No	2	92.5	73.6	18.9	78	Peripheral neuropathy, febrile neutropenia	21m	23m
Garcia-Sanz et al. [MC] (37),	2019	Prospective Phase I/II	66, 36	1	Bv + ESHAP	No	3	91	70	21	97	Neutropenia, Hypomagnesemia	71% at 30 months	91% at 30 months
Stamatoullas et al. [MC] (38),	2019	Prospective	42, 30	1	Bv + ICE	No	2	84.8	69.2	25.6	75	Neutropenia	69% at 1 yr	Not reached
Broccoli et al. [SC] (18),	2019	Prospective	40, 38	1	Bv + Be	No	6	84.2	78.9	5.3	92.1	Neutropenia	67.3% at 3 years	88.1% at 3 years
LaCasce et al. [MN] (39),	2020	Prospective	55, NR	1	Bv + Be	No	xx	92.5	73.6	18.9	75.5	Neutropenia, peripheral neuropathy	62.6% at 2 years	92% at 3 years
Advani et al. [MC] (40)	2021	Prospective Phase I/II	91, 34	1	Bv + Nivo	No	4	85	67	18	87	Neutropenia, peripheral neuropathy	77% at 3 yrs	93% at 3 yrs
Zagadailov et al. [MN] (21),	2017	Retrospective	196, NR	NR, multiple	Bv	Yes	7.5	80.6	45.4	35.2	NR	Neutropenia	27m	Not reached
Iannitto et al. [SC] (41)	2020	Retrospective	47, 34	2	Bv + Be	No	4	79	49	30	67	Neutropenia, GI, Neuropathy	18 months	72% at 2 years
Pinczés et al. [MC] (20)	2020	Retrospective	41, NR	1	Bv + Be	No	3	92.6	70.7	21.9	90	Neutropenia, Neuropathy, Infusion related reactions	62% at 2 years	93% at 2 years
**This study [SC]**	**2021**	**Retrospective**	**30, 30**	**3**	**Bv + Be**	**Yes**	**3**	**79**	**62**	**17**	**78**	**Neutropenia**	**58% at 3 years**	**75% at 3 years**

SC, Single centre; MC, Multi centre; MN, Multinational; Bv, Brentuximab Vedotin; Be, Bendamustine; ICE, Ifos + Cisplatin + Etoposide; ESHAP, Etoposide, cisplatin, methylpred, high dose ara-C; Nivo, Nivolumab; NR, Not reported/Not available; ORR, Overall response rate; CR, Complete response; PR, Partial response; EFS, Event free survival; OS, Overall survival.

Our dataset is possibly the first such study coming from a middle-income setting in R/R HL, as a bridge to HCT. The BBv regimen could be successfully delivered in an out-patient setting on most occasions. It was well tolerated with no discontinuations on account of infusion reactions or unmitigated grade III/IV toxicity. Though grade III/IV toxicities with skin rash, neuropathy, infections, and mucositis were reported, the toxicity of note was neutropenia which was fatal only in one patient. In a heavily treated subgroup of patients (median of 3 prior lines, including 4 AHCT and one alloHCT) with advanced stage (87%) and refractory disease (>60%) in many, an impressive ORR of 79% [CR: 62%] was achieved with a median of 3 cycles of BBv. Among them, 25 patients eventually underwent an HCT. The impact of achieving a complete metabolic response (CMR) post-BBv and post-HCT, on improved EFS is noteworthy and statistically significant. AHCT was the common strategy for consolidation. All patients with a plan to AHCT mobilized adequately. The lone ‘poor-mobilizer’ was a patient who had received 3 prior lines of therapy and mediastinal radiation therapy for tumor control. This patient had financial constraints, no related allogeneic donor and opted out of an alloHCT. In the heavily pretreated and refractory subset of patients, alloHCT was offered as consolidation therapy. It is only a matter of coincidence that among the alloHCT patients in this study, all of them underwent haploidentical donor alloHCT. This reflects an increasing relevance for haploidentical alloHCT as an alternative donor HCT strategy in middle-income countries where comprehensive matched unrelated marrow donor registries are in their early growth phase or are expensive to afford for many patients. The outcomes in the subgroup of our patients consolidated with haploidentical alloHCT is in line with emerging evidence that haploidentical alloHCT may be associated with improved outcomes in R/R HL (43). The impressive response rates with BBv spare many patients additional therapies like radiotherapy for disease control or consolidation. Only one patient in this entire cohort received additional radiation therapy. None of our patients received maintenance Bv, due to cost constraints.

Our study has some limitations. It is a retrospective study with its limitations, and this is not restricted to the nature of data collection from a hospital EMR. These would potentially affect our ability to determine cause and effect relationships. A limited number of patients, as in our study, compromise the ‘tests of significance’ to determine the true impact of an intervention. Further, our patient group is relatively young (median age = 30 years) with none or few manageable comorbidities. This limits the extrapolation of our study to all older adults. However, we believe this study is a true reflection of the real-world practice seen in many LMICs where cost-effectiveness of therapy is put to its true test when patients, with limited resources, are involved in out-of-pocket expenditures for their healthcare.

Access to novel therapeutics in India and other LMICs remains a challenge. Many strategies have been employed by LMICs to address this concern and this has been reviewed elsewhere (44). In India, access to new cancer drugs is commonly through pharma sponsored clinical trials (phase 2 and beyond), open label expanded access programs, and ‘named-patient licensed drug access’ program (through purchase or compassionate access mechanisms). Financial feasibility and regulatory pathways determine the time-to-local availability (in LMICs) of many cancer drugs approved in North America and Europe, despite comparable safety efficacy outcomes. Brentuximab vedotin (Adcetris^®^) is a case in point, and this study reflects that reality.

## Conclusion

Access to Brentuximab vedotin allowed our group to offer the novel out-patient based strategy using BBv in patients with R/R HL who could afford the costs of importing the drug. The regimen was well tolerated and safe. The outcome in our retrospective study compares favorably with published reports and demonstrates the feasibility of delivering such therapies in an LMIC setting. This study also helps us reflect on access related constraints of novel therapeutics in LMICs. This is driven primarily by the costs of the drug and in turn leads to poor uptake by patients who need them. This study also confirms the successful use of HCT as a consolidation strategy and provides pointers towards the growing popularity of haploidentical alloHCT as an alternative donor HCT strategy, in middle-income settings. The potential of this drug combination in a difficult-to-treat subset of HL patients and as a bridge to successful HCT, needs further testing in larger and randomized controlled trials.

## Data Availability Statement

The raw data supporting the conclusions of this article will be made available by the authors on a reasonable request to the corresponding author.

## Ethics Statement

Ethical review and approval was not required for the study on human participants in accordance with the local legislation and institutional requirements. Written informed consent for participation was not required for this study in accordance with the national legislation and the institutional requirements. Institutional Review Board (Ethics Committee) waiver No. EC/WV/TMC/23/21; Retrospective study with no patient personal identifiers.

## Author Contributions

Concept and design: VSR, RN, and MC. Literature search: VSR, RB, and VR. Clinical Management: VSR, RB, JK, SB, RS, AN, RA, MC, and RN. Laboratory and Imaging studies: IA, LZ, DD, NA, MP, DM, and JD. Data acquisition, data analysis: VR, RB, and VSR. Manuscript preparation: VSR, VR, and RB. Manuscript editing: RN and RS. Manuscript review: RN, VSR, RS, IA, MP, LZ, DD, DM, JD, RA, JK, SB, and MC. IA Guarantors: MC and RN. All authors contributed to the article and approved the submitted version.

## Conflict of Interest

VSR reports advisory fees (institutional) and non-financial Institutional support from PFIZER, Institutional grants and non-financial support from INTAS Pharmaceuticals, Institutional grants from NATCO Pharmaceuticals, Institutional grants from ROCHE, Institutional grants from BMS, Institutional grants and non-financial support from CIPLA Pharmaceuticals, Institutional grants from EMCURE, personal fees (institutional) from ASTRA ZENECA, non-financial institutional support from Dr. REDDY’s Laboratories, outside the submitted work. RN has received research grants, advisory board fees as well as Speaker fee from Cipla, Freisenius Kabi, Johnson and Johnson, Mylan, Novartis, and Dr Reddy’s Laboratory, outside the submitted work.

The remaining authors declare that the research was conducted in the absence of any commercial or financial relationships that could be construed as a potential conflict of interest.

## Publisher’s Note

All claims expressed in this article are solely those of the authors and do not necessarily represent those of their affiliated organizations, or those of the publisher, the editors and the reviewers. Any product that may be evaluated in this article, or claim that may be made by its manufacturer, is not guaranteed or endorsed by the publisher.
