# Supplementary material for: Relapsed Refractory Hodgkin Lymphoma and Brentuximab Vedotin-Bendamustine Combination Therapy as a Bridge to Transplantation: Real-World Evidence From a Middle-Income Setting and Literature Review
Source: Front Oncol. 2022 Jan 21;11:796270. doi: 10.3389/fonc.2021.796270 (PMC8814627; doi:10.3389/fonc.2021.796270)
Supplement: Supplementary file 1 [file DataSheet_1.docx]

**Table S1**

**Demographic and clinical characteristics of the R/R cHL patients who received treatment with other salvage regimen and underwent AHCT.**

| **N** | **18** |
| --- | --- |
| **Median age (range)** | **25 (16-50)** |
| **F (%)** | 7 (35) |
| **M (%)** | 11 (65) |
| **1^st^ Line (%)** |  |
| ABVD | 18 (100) |
| **2^nd^ line (%)** |  |
| DHAP | 4 (22) |
| AVD | 3 (16.5) |
| GDP | 9 (50) |
| ICE | 1 (5.5) |
| MINE | 1 (5.5) |
| **3^rd^ Line (%)** |  |
| DHAP/RDHAP | 4 (44) |
| GDP/RGDP | 3 (33) |
| IGEV | 1 (11) |
| GCD | 1 (11) |
| **Median salvage cycles (range)** | **3 (2-4)** |
| **Salvage regimen that achieved response prior to AHCT** |  |
| DHAP/RDHAP | 7 (39) |
| GDP/RGDP | 9 (50) |
| GCD | 1 (5.5) |
| IGEV | 1 (5.5) |
| **HCT type (%)** |  |
| AHCT (autologous) | 18 (100) |
| **Graft Source (%)** |  |
| PB | 18 (100) |
| **Conditioning (%)** |  |
| BEAM | 11 (61) |
| BeEAM (Be=Bendamustine) | 1 (5.5) |
| 1. BEAM | 5 (28) |
| GEM-BU-MEL | 1 (5.5) |
| **Stem cell moblilisation (%)** |  |
| GCSF alone | 5 (28) |
| GCSF + plerixafor | 13 (72) |
| **Median CD34+ cell dose x10^6^/kg (range)** | 5.82 (1.8-14.5) |
| **Median MNC x10^8^/kg (range)** | 10 (2.2-28.8) |
| **Median day of engraftment (range)** |  |
| Neutrophil | 10 (8-20) |
| Platelet | 10 (8-26) |
| **Pre-AHCT response status N=18 (%)** |  |
| CMR | 15 (83) |
| PR | 3 (17) |
| **Post-AHCT response status, N=16(%)** |  |
| CMR | 12 (75) |
| PD | 4 (25) |
| **Outcomes (%) (n=18)** |  |
| Relapse alone | 5(27.5) |
| Relapse and death | 1 (5.5) |
| Death | 1 (5.5) |
| **Regimen related toxicities (n=18) (%)** |  |
| Go | 1 (5.5) |
| G1 | 3 (16.5) |
| G2 | 4 (22) |
| G3 | 3 (16.5) |
| G4 | 7 (39) |

**Figure S1**

**Survival Curves for of the R/R cHL patients who received treatment with other salvage regimens.**

| 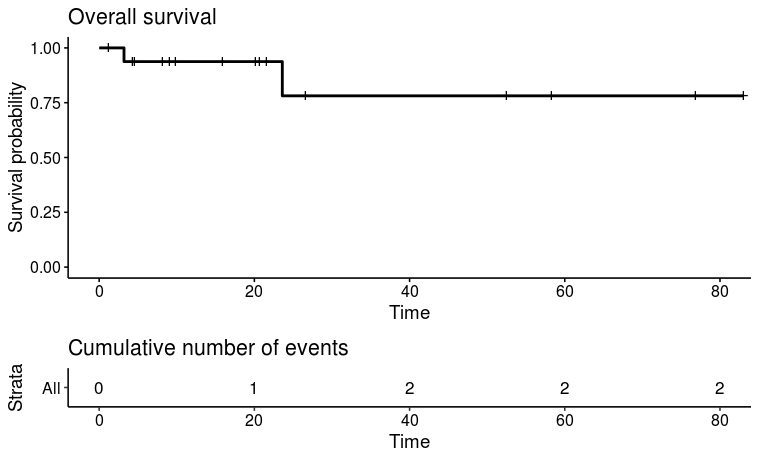 | 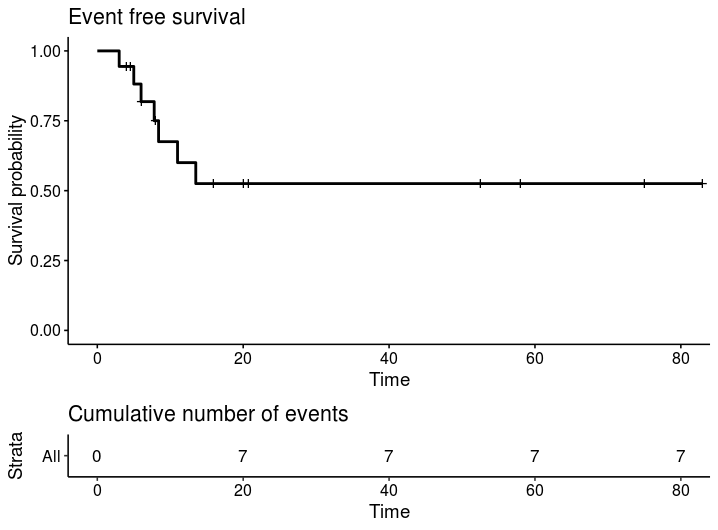 |
| --- | --- |
| (a). OS for other salvage regimens (n=18) | (b). EFS for other salvage regimens (n=18) |
| 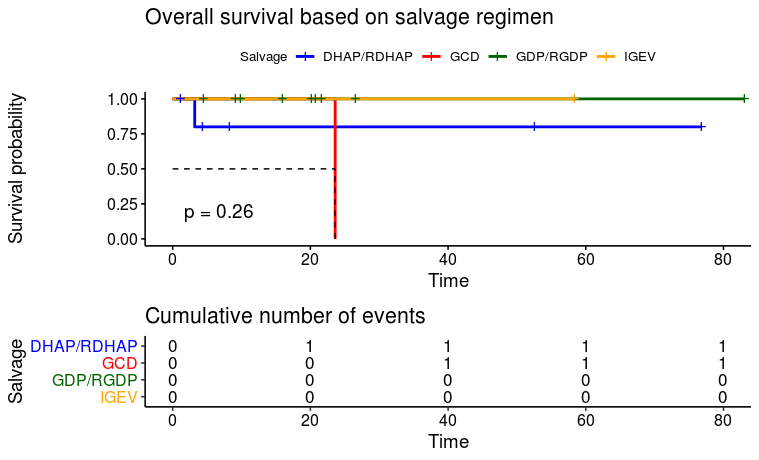 | 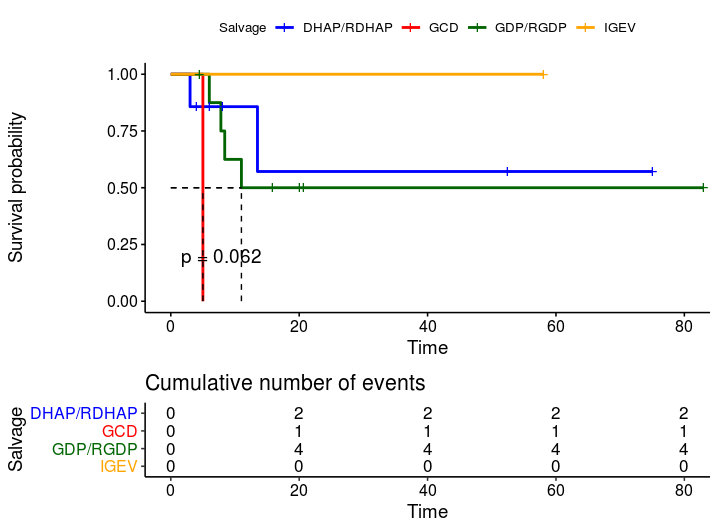 |
| (c). OS for patients stratified on salvage regimen (n=18) | (d). EFS for patients stratified on salvage regimen (n=18) |

**Figure S2.**

**Survival curves for BBv patients who underwent HCT.**

| 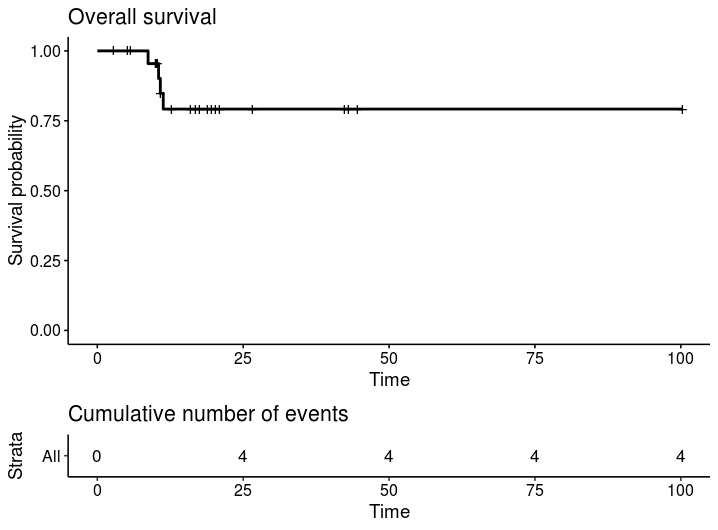 | 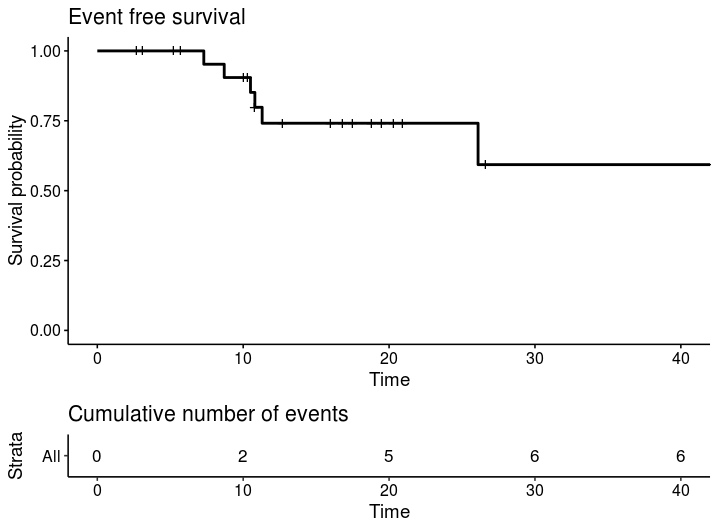 |
| --- | --- |
| (a).OS for BBv group undergone any transplant (n=25) | (b). EFS for BBv group undergone any transplant (n=25) |
| 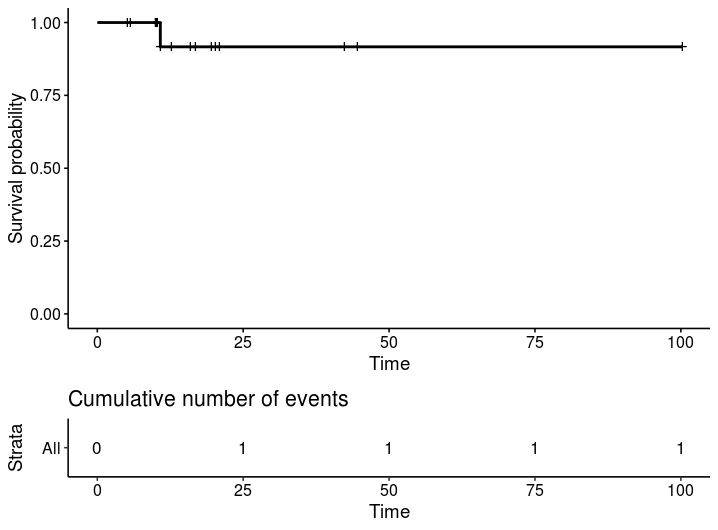 | 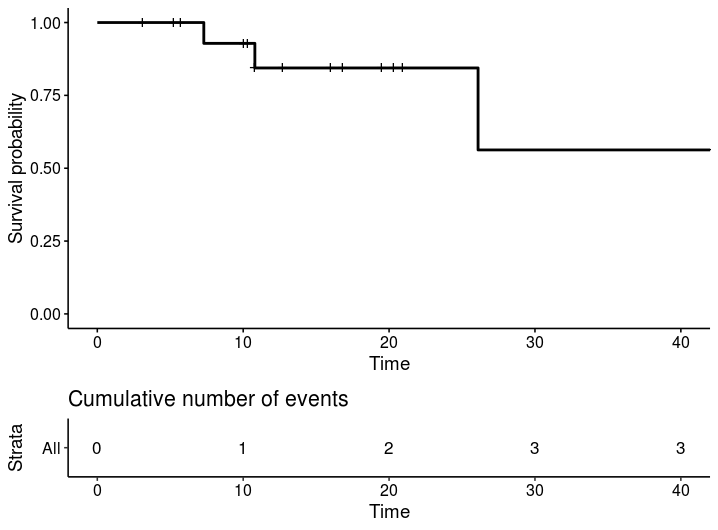 |
| (c). OS for BBv group undergone AHCT (n=17) | (d). EFS for BBv group undergone AHCT (n=17) |

**Figure S3.**

**Swimmer plot demonstrating time to response and events in BBv group (N=30).**

**
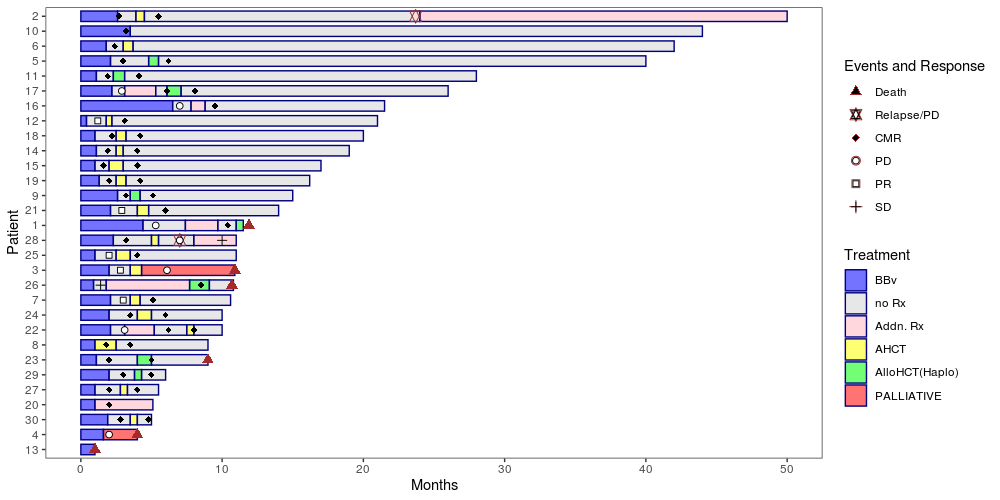
**
